# Skin-like Transparent Polymer-Hydrogel Hybrid Pressure Sensor with Pyramid Microstructures

**DOI:** 10.3390/polym13193272

**Published:** 2021-09-25

**Authors:** Kyumin Kang, Hyunjin Jung, Soojung An, Hyoung Won Baac, Mikyung Shin, Donghee Son

**Affiliations:** 1 Department of Electrical and Computer Engineering, Sungkyunkwan University (SKKU), Suwon 16419, Korea; zseqqq@gmail.com (K.K.); soojung2134@gmail.com (S.A.); hwbaac@skku.edu (H.W.B.); 2 School of Mechanical Engineering, Sungkyunkwan University (SKKU), Suwon 16419, Korea; hyunjin.jung33@gmail.com; 3 Department of Biomedical Engineering, Sungkyunkwan University (SKKU), Suwon 16419, Korea; 4 Department of Intelligent Precision Healthcare Convergence, Sungkyunkwan University (SKKU), Suwon 16419, Korea; 5 Center for Neuroscience Imaging Research, Institute for Basic Science (IBS), Suwon 16419, Korea; 6 Department of Superintelligence Engineering, Sungkyunkwan University (SKKU), Suwon 16419, Korea

**Keywords:** e-skin, electronic skin, soft pressure sensor, soft device, transparent, skin-like hybrid sensor

## Abstract

Soft biomimetic electronic devices primarily comprise an electronic skin (e-skin) capable of implementing various wearable/implantable applications such as soft human–machine interfaces, epidermal healthcare systems, and neuroprosthetics owing to its high mechanical flexibility, tissue conformability, and multifunctionality. The conformal contact of the e-skin with living tissues enables more precise analyses of physiological signals, even in the long term, as compared to rigid electronic devices. In this regard, e-skin can be considered as a promising formfactor for developing highly sensitive and transparent pressure sensors. Specifically, to minimize the modulus mismatch at the biotic–abiotic interface, transparent-conductive hydrogels have been used as electrodes with exceptional pressing durability. However, critical issues such as dehydration and low compatibility with elastomers remain a challenge. In this paper, we propose a skin-like transparent polymer-hydrogel hybrid pressure sensor (HPS) with microstructures based on the polyacrylamide/sodium-alginate hydrogel and p-PVDF-HFP-DBP polymer. The encapsulated HPS achieves conformal contact with skin due to its intrinsically stretchable, highly transparent, widely sensitive, and anti-dehydrative properties. We believe that the HPS is a promising candidate for a robust transparent epidermal stretchable-skin device.

## 1. Introduction

Inspired by human tissues, various approaches have been proposed to mimic the body for soft devices such as bio-integrated electronics [1,2], electronic skins (e-skins) [3,4,5,6], and human-machine interface devices [7,8]. In particular, soft e-skin, inspired by human skin, is used in robotics [9,10], skin-attachable electronics [11,12], and prosthetics [13,14,15] for biomimetic features such as stretchability, mechanical stability, and tactile sensing properties. For instance, stretchable and conductive polymers such as Ag nanowires [16], Ag flakes [17], carbon nanotubes (CNT) [18,19], PEDOT: PSS [20,21], nanomembranes [22,23], and liquid metals [24,25] have been used as e-skins. These polymers can monitor external stimuli such as strains, pressures, and temperatures and are mechanically robust and sufficiently stable in various environments to convert the stimuli into electrical signals. However, polymer-based e-skin devices have a relatively high moduli and low stretchability, as compared to skin.

Hydrogels, unlike polymers, have relatively low moduli [26], intrinsic stretchability [27], biocompatibility [28], and transparency [29]. The elastic modulus of a hydrogel is in the kilopascal range, which is lower than that of other compatible polymers owing to the presence of water. The aggregation of the non-toxic cross-linked chain network of a hydrogel can help withstand mechanical strains up to approximately 1000%, while also being biocompatible. In addition, conductive hydrogels, such as conductive polymers, can help monitor external stimuli and convert them into electrical signals. However, hydrogel-based e-skin devices suffer from the limitations of low robustness and dehydration.

**Figure 1:**
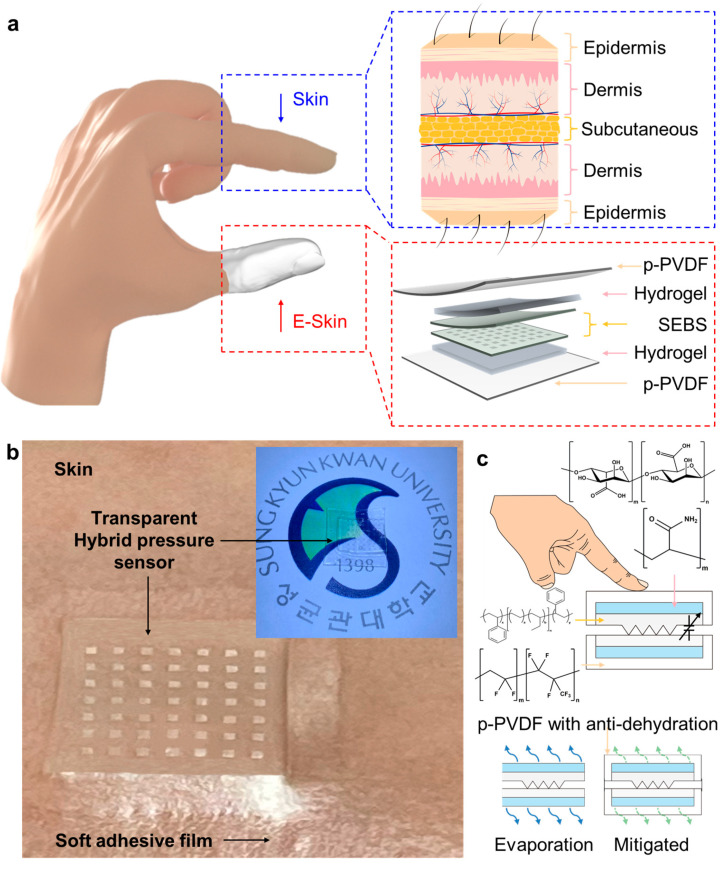
(**a**) Overall schematic illustration of the transparent hybrid soft pressure sensor. (**b**) Photograph of the transparent polymer-hydrogel hybrid pressure sensor. Inset image shows the better transparency of the sensor. (**c**) Schematics for the underlying mechanism of pressure sensing based on the sensor and the materials strategy of long-term stable pressure sensing using p-PVDF-based self-bonding assembly.

Because polymers and hydrogels have complementary properties, it is desirable to combine them into polymer-hydrogel hybrid structures, such as e-skins, that can improve performance or overcome existing problems. Various strategies have used polymer-hydrogel hybrid structures for realizing transparency, stable monitoring, robustness, and softness [30,31,32,33]. This skin-mimetic hybrid structure has an intrinsically stretchable hydrogel layer with a modulus similar to that of the dermis of the skin [34,35]. The hybrid structure also has an external polymer layer with a modulus similar to that of the epidermis, which prevents dehydration of the dermis and relieves external stimuli via encapsulation [36,37]. Figure 1 depicts the overall schematics of a skin-like polymer-hydrogel hybrid soft pressure sensor.

Herein, we propose a skin-like polymer-hydrogel hybrid pressure sensor (HPS) using pressure-sensitive microstructures. Our HPS device uses intrinsically stretchable and transparent hydrogel layers. The HPS device was also encapsulated with a polarized-poly (vinylidene fluoride-co-hexafluoropropylene) (PVDF-HFP)-dibutyl phthalate (DBP) polymer layer to realize a transparent and intrinsically stretchable device [34]. The pressure sensor applied to the skin-like polymer-hydrogel hybrid structure exhibited anti-dehydrative and relatively robust properties.

## 2. Materials and Methods

### 2.1. Fabrication of a Silicon Pyramid Mold for Pressure-Sensitive Microstructures

**Figure 2:**
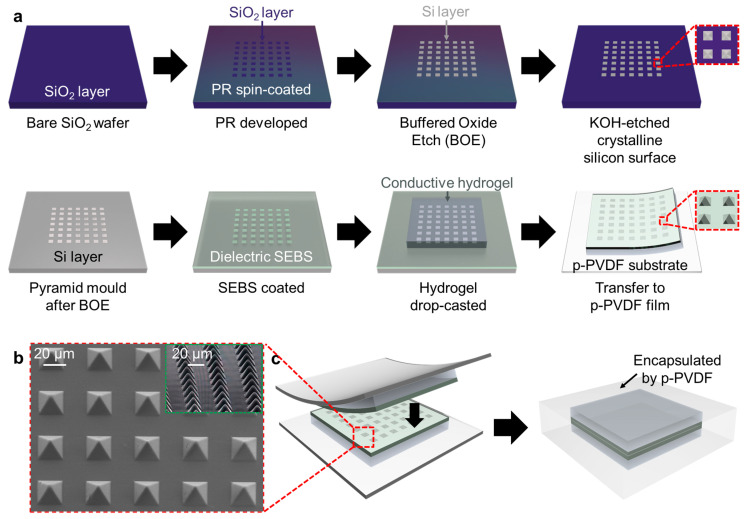
(**a**) Schematics for the fabrication processes of a silicon pyramid mold for the microstructure and skin-like polymer-hydrogel hybrid soft pressure sensor. (**b**) SEM images of pressure-sensitive pyramid microstructures. (**c**) Schematic for allowing the skin-like polymer-hydrogel hybrid soft pressure sensor to be air-stable using p-PVDF encapsulation films.

The polymer layer was fabricated using a pressure-sensitive microstructure, as schematically illustrated in Figure 2a. The SEBS (TuftecTM H1062, Asahi Kasei Co., Tokyo, Japan) pyramids were fabricated using a wet-etched silicon wafer substrate. A silicon wafer with a 300 nm thermally grown oxide layer was prepared with dimensions of 30 mm × 3 mm. At the beginning of the fabrication process, the silicon wafer was immersed in acetone and isopropyl alcohol to clean the surface of the substrate and was then surface-treated with bath ultrasonication in deionized (DI) water for 5 min. Subsequently, the wafer was subjected to reactive ion etching (RIE; PlasmaLab system 80 RIE, Oxford Instruments, Abingdon, UK) with O_2_ 20 sccm at a pressure of 100 mTorr with 300 W RF power for 5 min. After surface cleaning treatment, a positive photoresist (S1805, Merck KGaA, Darmstadt, Germany) was spin-coated with a spin-coater (SPIN-3000D, MIDAS SYSTEM Co., Ltd., Daejeon, Korea) at 3000 rpm for 30 s. The PR-coated wafer was prebaked at 110 °C for 1 min, and photolithography was performed using a mask aligner (MDA-400M, MIDAS SYSTEM Co., Ltd., Daejeon, Korea) with 200 mW RF power for 10 s to draw square patterns on the substrate, which was to be etched into a pyramid structure. A developer (MIF-300, Merck KGaA, Darmstadt, Germany) was used to remove the exposed PR, resulting in square patterns. Thereafter, the oxide layer was etched using a 6:1 buffered oxide etch (BOE;;Sigma-Aldrich, Burlington, MA, USA) solution, followed by bath ultrasonication for 5 min. Subsequently, the wafer was immersed in acetone to remove the undeveloped PR. The substrate silicon was then etched with 30% potassium hydroxide (KOH; Sigma-Aldrich, Burlington, MA, USA) at 60 °C for 3 h and 30 min. After the pyramid pits were fully generated via KOH etching, the wafer was ultrasonicated for 15 min, and the BOE was etched again to remove the SiO_2_ residual. The fabricated silicon cavities were treated with vapor phase 1H,1H,2H,2H-perfluorodecyltrichlorosilane (Sigma-Aldrich, Burlington, MA, USA) and baked at 120 °C for 1 h to lower the surface energy and the adhesion force between SEBS and the silicon mold. The SEBS solution (300 mg/mL in toluene) was spin coated on the fabricated wafer and the treated bare wafer and then cured overnight. Figure 2b shows the scanning electron microscopy (SEM) images of the pyramid-shaped microstructure of the SEBS dielectric layer. 

### 2.2. Fabrication of Skin-like polymer-hydrogel Hybrid Pressure Sensor

A capacitive flexible pressure sensor was fabricated, as schematically illustrated in Figure 2a–c. To connect the hydrogel network, sodium alginate (SA; Sigma-Aldrich, Burlington, MA, USA) and acrylamide (AM; Sigma-Aldrich, Burlington, MA, USA) were dissolved in deionized water. After stirring evenly, *N*,*N*-methylenebisacrylamide (MBA; Sigma-Aldrich, Burlington, MA, USA) and *N*,*N*,*N*,*N*-tetramethyl ethylenediamine (TEMED; Sigma-Aldrich, Burlington, MA, USA) were added to the DI water solution as a crosslinker and crosslinking accelerator for polyacrylamide. Ammonium persulfate (APS; Sigma-Aldrich, Burlington, MA, USA) was added as an initiator for acrylamide polymerization. After stirring again, the DI water solution was poured onto the SEBS dielectric layer. The SEBS-coated wafer and the pre-solution of the hydrogel were thermally baked at 60 °C. After curing, the double-layer film was peeled off from the wafer and transferred to the p-PVDF-HFP-DBP film. Subsequently, a skin-like polymer-hydrogel HPS was prepared by repeating the same process and overlapping the two samples. Because the p-PVDF-HFP-DBP layer is highly cohesive and self-healable, the inner hydrogels were encapsulated to prevent dehydration.

## 3. Results

### Characteristics of polymer-hydrogel Hybrid Pressure Sensor

**Figure 3:**
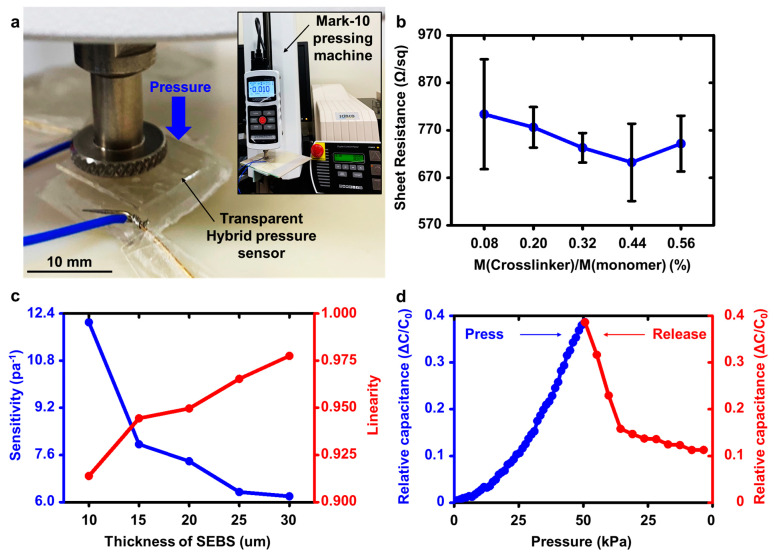
(**a**) Image of pressure sensing operation using a pressing machine (Mark-10). (**b**) Plot of sheet resistance of five tough conducting hydrogels as a function of with different crosslinking ratios. (**c**) Sensitivity (blue) and the corresponding linearity (red) of the pressure sensor versus thickness of SEBS. Variations in initial capacitance and sensitivity with respect to the thickness of SEBS for determining the range of use. (**d**) Pressure sensing profile of the pressure sensor when pressed and released.

To characterize the polymer-hydrogel hybrid structure of the pressure sensor fabricated, as discussed in Section 2, a press test was performed using a motorized force tester device (ESM303, Mark-10 Co., New York, NY, USA) at a humidity of about 70% and a temperature of 23 °C. Figure 3a illustrates the press test of the prototype pressure sensor and the experimental setup. 

Figure 3b shows the design rules for determining the weight ratio of the MBA. Here, we confirmed that the electrical conductivity of the hydrogels does not significantly affect the sensing capability of the pressure sensor. However, if our pressure sensor is integrated with other electronic components, the electrical conductivity issue of the hydrogel issue becomes crucial due to resistance-capacitance delays. For this reason, we adopted the conducting hydrogel with the lowest sheet resistance (a ratio of MBA crosslinker to monomer) is 0.44% (Figure 3b). Specifically, the sheet resistance of a hydrogel depends on the size of the polymeric space network. If the amount of MBA is excessively small, the spaces in the polymeric network are not sufficiently formed, leading to the low electrical conductivity of the hydrogel. In contrast, if the amount of MBA is excessively high, the space in the polymeric network becomes small (Supplementary Figure S1) [35,38,39].

Figure 3c shows the design rule for determining the optimal thickness of the SEBS dielectric layer. The sensitivity, linearity, hysteresis, and initial capacitance values depend on the SEBS thickness (Supplementary Figure S2). As the thickness of the SEBS dielectric layer became thicker, the initial capacitance value and sensitivity of the pressure sensor were decreased; however, the linearity and hysteresis of the pressure sensor were improved. Conversely, as the thickness of the SEBS dielectric layer became thinner, the initial capacitance value and sensitivity of the pressure sensor were increased; however, the linearity and hysteresis of the pressure sensor were degraded. Such a result originates from viscoelasticity of the SEBS film. From the asymmetric trends, we chose the optimal SEBS thickness of 15 μm to meet a simultaneous requirement regarding sensitivity, linearity, hysteresis and initial capacitance (Supplementary Figure S3). Such critical parameters should be carefully chosen in determining electrical performance of the skin-like devices. The sensitivity plays a crucial role in evaluating the performance of the pressure sensor. We fabricated HPS with a relatively high sensitivity using pressure-sensitive microstructures. For this reason, a SEBS thin dielectric film with a high sensitivity (7.7 Pa^−1^) was selected. The linearity and hysteresis are also important parameters in the sensing properties. Low linearity and hysteresis led to undesired degradation in the measurement methodology, which requires an additional feedback loop system or a computing algorithm [40,41,42]. Figure 3d shows the relative capacitance–pressure curve depicting the sensitivity of the pressure sensor. The diameter of the press cylinder was 10 mm, and the pressing and releasing speeds were 3 mm/min. When we applied a gentle pressure of 50 kPa to the pressure sensor, the linear sensing profile was confirmed. This sensing range was much wider than those of previous pyramidal structure-based sensors [15]. However, its linearity was slightly distorted when released. This result was also mainly due to the fatigue of the SEBS microstructures. Such a challenge can be mitigated by applying elastic materials with higher modulus values.

**Figure 4:**
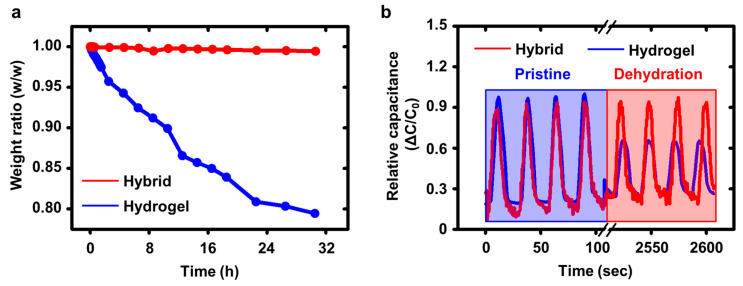
(**a**) Comparison of the pressure sensing durability of the HPS to that of HPS without HFP encapsulation. (**b**) Pressing cyclic data of the HPS.

In addition to the sensing performance of the pressure sensor, air-stability was addressed in Figure 4. Generally, the hydrogel composed of water and polymer networks is vulnerable to air environment due to a dehydration issue. To overcome the challenge, we used the p-PVDF film, which is capable of homogeneous interface between two individual layers without any external stimuli due to dipole–dipole interaction. Using the p-PVDF films, we encapsulated the pressure sensor. First, we verified the air stability of the p-PVDF-based pressure sensor (Figure 4a). As expected, the pressure sensor without the p-PVDF encapsulation layers was gradually degraded. However, the encapsulated sensor showed the exceptional stability. This result fully supported our assumption. Figure 4b showed the pressing cyclic data (up to 100 cycles) of the pressure sensors with (red, hybrid) and without (blue, bare hydrogel) encapsulation layers. The p-PVDF encapsulation allowed the pressure sensor to be more robust than before.

## 4. Discussion and Conclusions

In this work, we demonstrated a skin-like polymer-hydrogel HPS with pressure-sensitive microstructures that can complement the properties of polymers and hydrogels through hybrid structures. The HPS device was intrinsically stretchable and highly transparent; it could address the dehydration issues due to p-PVDF-HFP-DBP polymer encapsulation. Additionally, the performance of the HPS was controlled by optimizing the thickness of the dielectric layer and the ratio of acrylamide in the hydrogel layer. Based on the self-bonding assembly of p-PVDF films, we demonstrated that the pressure sensing capability of our HPS device is very durable compared with those of previous hydrogel-based sensors without encapsulation. Thus, we believe that HPS is a promising candidate for long-term biomimetic e-skin devices.

## Data Availability

The data presented in this study are available in this article.

## Supplementary Materials

The following are available online at https://www.mdpi.com/article/10.3390/polym13193272/s1, Figure S1: (a) The chemical structures of the PAAm hydrogel, Polyacrylamide and *N*,*N*-methylenebisacrylamide. (b)Schematic diagrams of polymeric network structure with different crosslinker/monomer proportion. As the amount of MBA increases, the polymeric network space becomes denser, Figure S2: (a) Initial capacitance (blue) and hysteresis (red) of the pressure sensor versus thickness of SEBS. (b) Hysteresis calculation method, Figure S3: (a) Raw data (blue) and Regression of raw data (red) of the pressure sensor which has a 15 μm thickness of SEBS. (b) Linearity calculation method.
