# Supplementary material for: Skin-like Transparent Polymer-Hydrogel Hybrid Pressure Sensor with Pyramid Microstructures
Source: Polymers (Basel). 2021 Sep 25;13(19):3272. doi: 10.3390/polym13193272 (PMC8513041; doi:10.3390/polym13193272)
Supplement: Supplementary file 1 [file polymers-13-03272-s001.zip › polymers-1383041-supplementary.pdf]

Supplementary materials for

"Skin-like transparent polymer–hydrogel hybrid pressure sensor with pressure sensitive microstructures"

Kyumin Kang[1], Hyunjin Jung[2], Soojung An[1], Hyoung Won Baac[1], Mikyung Shin[3,4,5,*] and Donghee Son[1,5,6,*]

[1] Department of Electrical and Computer Engineering, Sungkyunkwan University (SKKU), Suwon 16419, Korea; zseqqq@gmail.com (K.K.); soojung2134@gmail.com (S.A.); hwbaac@skku.edu (H.W.B)
[2] School of Mechanical Engineering, Sungkyunkwan University (SKKU), Suwon 16419, Korea; hyunjin.jung33@gmail.com (H.J.)
[3] Department of Biomedical Engineering, Sungkyunkwan University (SKKU), Suwon 16419, Korea; mikyungshin@g.skku.edu (M.S.)
[4] Department of Intelligent Precision Healthcare Convergence, Sungkyunkwan University (SKKU), Suwon 16419, Korea.
[5] Center for Neuroscience Imaging Research, Institute for Basic Science (IBS), Suwon 16419, Korea
[6] Department of Superintelligence Engineering, Sungkyunkwan University (SKKU), Suwon 16419, Korea

* Correspondence: daniel3600@g.skku.edu; Tel./Fax: +82-312907696

Supplementary Figure S1

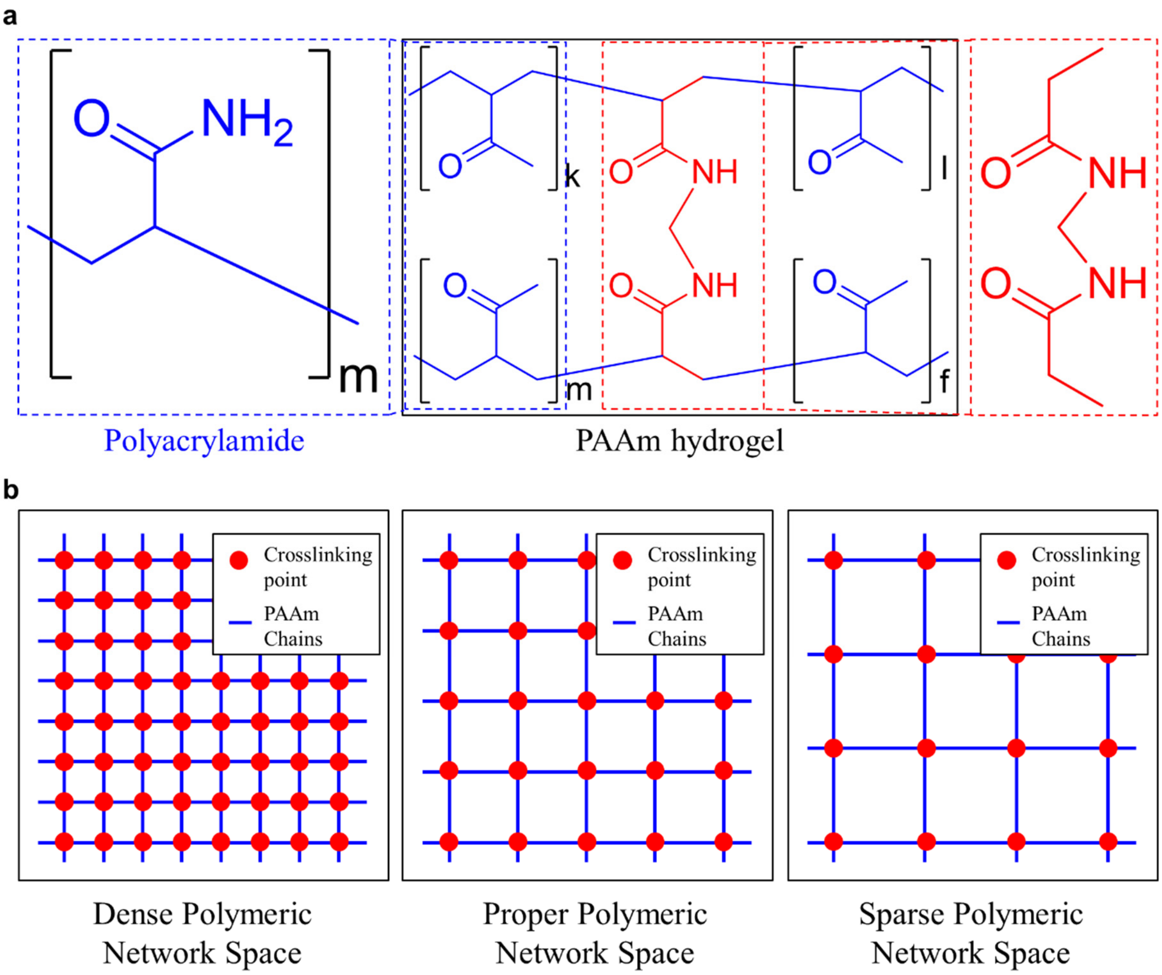

Figure S1. a) The chemical structures of the PAAm hydrogel, Polyacrylamide and N,N-methylenebisacrylamide. b)Schematic diagrams of polymeric network structure with different crosslinker/monomer proportion. As the amount of MBA increases, the polymeric network space becomes denser.

Supplementary Figure S2

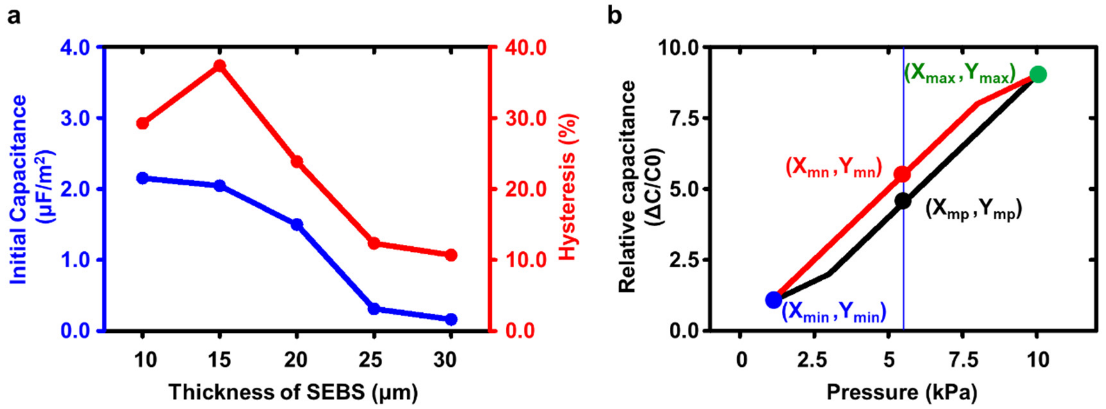

Figure S2. a) Initial capacitance (blue) and hysteresis (red) of the pressure sensor versus thickness of S EBS. b) Hysteresis calculation method.

Supplementary Figure S3

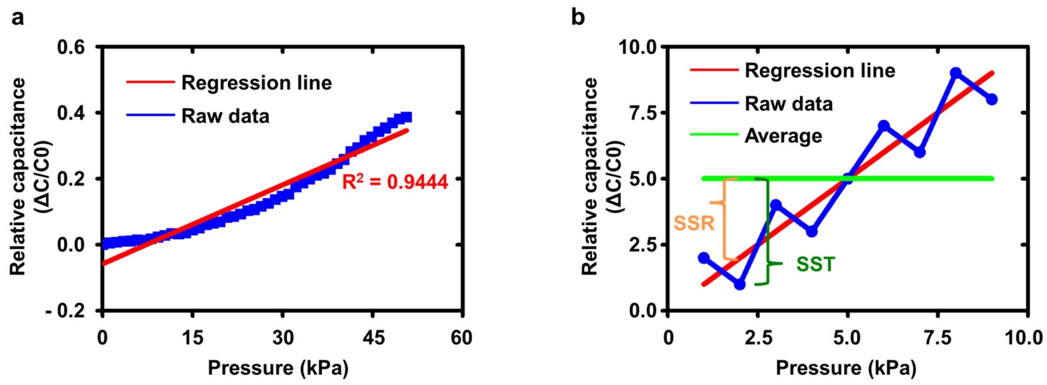

Figure S3. a) Raw data (blue) and Regression of raw data (red) of the pressure sensor which has a 15 μ m thickness of SEBS. b) Linearity calculation method.

Supplementary note 1:

The linearity and hysteresis of pressure sensor is defined as the coefficient of determination value of the regression line and ratio of deviation of the median to the pressure range. The coefficient of determination value and hysteresis in pressure sensor is defined as:

Sum of squared total (SST)

$$SST = \sum_{i=1}^{n} (y_i - \overline{y})^2$$

Sum of squared residual (SSR)

$$SSR = \sum_{i=1}^{n} (y_i - \hat{y}_i)^2$$

Coefficient of determination

$$R^2 = 1 - \frac{SSR}{SST}$$

$$Hysteresis = \left| \frac{\left(Y_{mn} - Y_{mp}\right)}{\left(Y_{\max} - Y_{\min}\right)} \right| \times 100\%$$
